# Socioeconomic and Other Risk Factors for Retear after Arthroscopic Surgery for Nontraumatic Rotator Cuff Tear

**DOI:** 10.3390/medicina60040640

**Published:** 2024-04-17

**Authors:** Jung Sub Lee, Kuen Tak Suh, Won Chul Shin, Jung Yun Bae, Tae Sik Goh, Sung Won Jung, Min-Hyeok Choi, Suk-Woong Kang

**Affiliations:** 1 Department of Orthopedics, Research Institute for Convergence of Biomedical Science and Technology, Pusan National University Hospital, Pusan National University School of Medicine, Busan 49241, Republic of Korea; jungsublee@pusan.ac.kr (J.S.L.); taesikgoh@pusan.ac.kr (T.S.G.); 2 Department of Orthopedics, Sehung Hospital, Busan 47250, Republic of Korea; kuentak@pusan.ac.kr; 3 Department of Orthopedics, Research Institute for Convergence of Biomedical Science and Technology, Pusan National University Hospital, Pusan National Yangsan University School of Medicine, Yangsan 50612, Republic of Korea; dreami3e5t@pusan.ac.kr (W.C.S.); nari1006@hanmail.net (J.Y.B.); cbcb1kr@naver.com (S.W.J.); 4 Department of Preventive and Occupational Medicine, Research Institute for Convergence of Biomedical Science and Technology, Pusan National University Yangsan Hospital, Pusan National University School of Medicine, Yangsan 50612, Republic of Korea; come2mh@pusan.ac.kr; 5 Office of Public Health Service, Pusan National University Yangsan Hospital, Pusan National University School of Medicine, Yangsan 50612, Republic of Korea

**Keywords:** nontraumatic rotator cuff tear, retear, arthroscopic surgery, risk factors, socioeconomic factors

## Abstract

*Background and Objectives:* Few studies have investigated the socioeconomic factors associated with retear after rotator cuff repair. This study aimed to identify the risk factors, including socioeconomic factors, for rotator cuff retear in patients who underwent arthroscopic rotator cuff repair. *Materials and Methods:* This retrospective study included 723 patients diagnosed with full-thickness rotator cuff tears who underwent arthroscopic rotator cuff repair from March 2010 to March 2021. The outcome variable was rotator cuff retear observed on postoperative magnetic resonance imaging or ultrasonography. Sex, age, obesity, diabetes, symptom duration, and tear size were the independent variables. Socioeconomic variables included occupation, educational level, type of medical insurance, and area of residence. We compared patients with and without retear and estimated the effects of the independent factors on retear risk. *Results:* The mean age of the patients, symptom duration, and tear size were 62.4 ± 8.0 years, 1.8 ± 1.7 years, and 21.8 ± 12.5 mm, respectively. The age, type of medical insurance, diabetes, tear size, and symptom duration differed significantly between patients with and without retearing (*p* < 0.05). Age, occupation, type of medical insurance, diabetes, initial tear size, and symptom duration significantly affected the risk of retear. Patients who performed manual labor had a significantly higher retear rate (*p* = 0.005; OR, 1.95; 95% CI, 1.23–3.11). The highest retear risk was seen in patients with Medicaid insurance (*p* < 0.001; OR, 4.34; 95% CI, 2.09–9.02). *Conclusions:* Age, initial tear size, and symptom duration significantly affect retear risk after arthroscopic rotator cuff repair. Occupation and type of medical insurance were also risk factors for retear. Socioeconomically vulnerable patients may be at a greater risk of retear. Proactive efforts are required to expand early access to medical care.

## 1. Introduction

Surgical results for rotator cuff tears are generally good; however, the high frequency of retears is a cause for concern [1]. Thus, several studies on rotator cuff retears have been conducted [1,2,3,4,5,6,7,8]. Rotator cuff retears are caused by various factors, such as patient, structural, and surgical factors. According to several previous studies, age, diabetes, hyperlipidemia, body mass index (BMI), and smoking are patient-related risk factors for retear [9,10,11,12,13,14]. Additionally, initial tear size, combined subscapularis tear, fatty degeneration, and fatty infiltration are considered independent structural risk factors for retear [1,15]. Moreover, the operator’s experience and the surgical method used also affect the risk of retear; in this regard, several surgical methods have been studied [1,8,15].

The initial size of a rotator cuff tear is associated with clinical outcomes, as well as with the risk of retear [1,6]. Therefore, in our previous study, we analyzed the factors influencing initial tear size [16]. These factors included age, duration of symptoms, type of medical insurance, region of residence, and occupation. Through this study, we were able to recognize that tear size, an important risk factor for rotator cuff retear, is influenced by socioeconomic factors.

Socioeconomic factors are important in various diseases [16,17]. Depending on the socioeconomic level, the prevalence of disease, delayed diagnosis, treatment, and patient experience with diagnosis and treatment vary greatly [18,19,20,21]. Barrack et al. [22] demonstrated poorer outcomes in patients with an annual income of <USD 25,000 in an analysis of functional outcome and patient satisfaction stratified according to socioeconomic factors after total knee arthroplasty. Similarly, Butler et al. [23] found that race, educational level, and household income were important risk factors for reduced functional status and satisfaction after total hip arthroplasty. Both studies reported that socioeconomic factors were as important as surgical factors in determining postoperative functional outcomes.

However, there is a lack of studies investigating the socioeconomic risk factors for retear after ARCR [2]. Cho et al. [3] reported poorer early clinical outcomes in women after ARCR. Moreover, Razmjou et al. [4] reported that gender disparity affects the mechanism of injury, perceived disability, medication consumption, referral pattern, and waiting time for surgery. In a systematic review of social determinants of health that are related to rotator cuff surgery, occupation, type of medical insurance, education level, race and ethnicity, area of residence, and gender were analyzed as socioeconomic factors [2]. This review reported that social determinants of health affect clinical outcomes, complication rate, and the rate of failed repair after rotator cuff surgery.

Considering the lack of information on socioeconomic factors associated with retear after ARCR, this study aimed to determine the risk factors, including socioeconomic factors, for rotator cuff retear in patients who underwent ARCR.

## 2. Materials and Methods

This study was approved by the Institutional Review Board of Pusan National University Yangsan Hospital (IRB No. 05-2021-185).

### 2.1. Study Population

**Figure 1:**
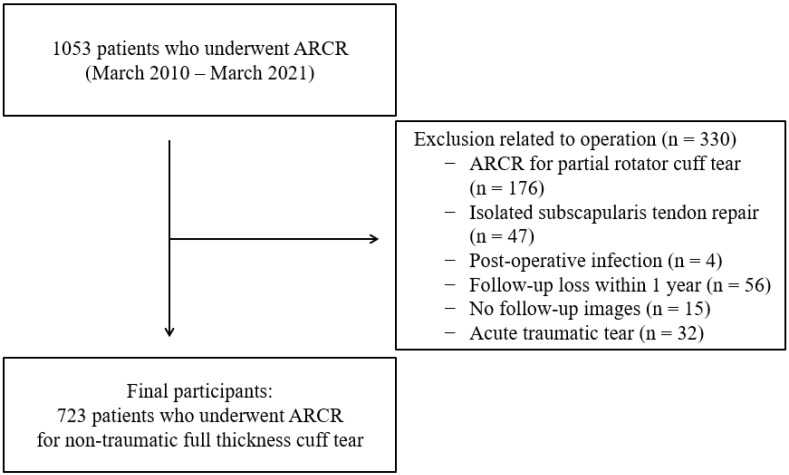
Diagram of the participants.

This retrospective study was conducted between March 2010 and March 2021 in patients diagnosed with rotator cuff tears who underwent ARCR. The authors searched the ICD-10 code (M751) and surgical codes (N0935, N0936, N0937, and N0938) to identify patients who underwent ARCR. In total, 1053 patients with rotator cuff tears were identified. The inclusion criteria were as follows: (1) history of ARCR for nontraumatic rotator cuff tear, (2) follow-up period ≥1 year, and (3) retear diagnosed by magnetic resonance imaging (MRI) or ultrasonography within 1 year. The exclusion criteria were as follows: (1) history of ARCR for partial rotator cuff tear, (2) isolated tear of the subscapularis tendon, (3) postoperative infection, (4) acute traumatic rotator cuff tear (definite trauma history and MRI findings [bone bruise, hemarthrosis, and edema in the rotator cuff or muscle]), (5) lack of a 1-year follow-up period, and (6) MRI or ultrasonography examination not performed to confirm a retear within 1 year. Finally, 723 patients who underwent ARCR for a rotator cuff tear were retrospectively analyzed (Figure 1).

### 2.2. Variables

The outcome variable was rotator cuff retear confirmed by postoperative MRI or ultrasonography. All patients had regular outpatient visits (at 1, 3, 6, 9, and 12 months, and once a year thereafter), and after 3 months, the diagnosis of retear was established by MRI or ultrasonography by a shoulder orthopedic radiologist with more than 10 years of experience. Retears were classified as type IV or V using the Sugaya classification (type IV: small, high-signal-intensity lesion, which suggests a small, full-thickness tear; type V: retorn, retracted tendon) [24].

After reviewing the literature, independent variables affecting rotator cuff retear were selected [4,5,6,7,8,9,10]. Sex, age, obesity (BMI ≥ 25 kg/m^2^), and diabetes (fasting blood sugar level ≥ 126 mg/dL or diabetic medication use) were selected as patient-related independent variables. The duration (in years) of symptoms, such as shoulder pain, weakness, and joint stiffness, was also investigated.

As a structural factor, tear size was measured via preoperative MRI and selected as an independent variable. Shoulder MRI was performed using 1.5-T (MAGNETOM Avanto, Siemens Healthineers, Erlangen, Germany) and 3-T (MAGNETOM Verio, Skyra, and VIDA, Siemens Healthineers) MRI systems, which included axial, oblique coronal, and oblique sagittal T1-weighted and fat-suppressed T2-weighted fast spin-echo sequences (FSEs). Mediolateral measurements from coronal oblique fat-suppressed T2-weighted FSE and anteroposterior measurements from sagittal oblique fat-suppression T2-weighted FSE were referenced to measure the size of the supraspinatus/infraspinatus tendon tear. The larger of the two values was considered the tear size.

The socioeconomic variables were occupation (non-manual or manual labor), educational level (≤middle school or ≥high school), area of residence (urban or rural), and type of medical insurance (National Health Insurance [NHI] or Medicaid). Occupations were classified based on the current job if currently employed and the longest job held if retired. Housewives were included in the non-manual labor group. The survey was conducted based on the Korean standard occupational classification system, and occupational groups based on repetitive physical work were defined as manual labor. Most Koreans are covered by the NHI, and the insurance fund is run by the Korean government based on the national fund and the NHI subscriber’s monthly premium payment. NHI subscribers pay only a portion of hospitalization and surgery costs. Meanwhile, individuals with lower socioeconomic status are enrolled in the Medicaid system. In 2019, Medicaid beneficiaries accounted for 2.9% of the total South Korean population [25]. Thus, insurance items, along with the right to basic livelihood benefits, are indicators of Korea’s socioeconomic level and are often used as factor variables in health inequality studies [18,26,27]. Areas of residence were divided into urban for *dong* addresses and rural for *eup* or *myeon* addresses by referring to medical records. This classification has previously been used in studies of health disparities between urban and rural areas and the effects of area on health [28,29].

### 2.3. Statistical Analysis

The patients were divided into two groups according to the occurrence of retear after ARCR. To compare the two groups, an independent *t*-test and chi-square test were performed for continuous and categorical data, respectively. A multivariate logistic regression model was used to determine the best predictor of retear after ARCR. Odds ratios (with 95% confidence intervals [CIs]) for retear were calculated for each parameter in the multivariate analysis. All statistical analyses were performed using Stata/MP version 17.0 (Stata Corporation, College Station, TX, USA).

## 3. Results

### 3.1. Baseline Data

Table 1 presents the general characteristics of the participants. Among the 723 patients, 401 were female (55.5%). The mean age of the patients was 62.4 ± 8.0 years. The group aged 60–69 years had the most patients (322), followed by the group aged 50–59 years. The mean age of patients in rural areas was 63.9 ± 8.2 years, while that in urban areas was 61.8 ± 7.8 years, showing a significant difference (*p* = 0.033). There was no difference between the sexes at the time of surgery. The mean symptom duration was 1.8 ± 1.7 years, and the mean tear size was 21.8 ± 12 mm. There were 137 patients with small-size tears within 1 cm, 434 patients with medium-size tears within 1–3 cm, and 152 patients with large-to-massive-size tears over 3 cm.

### 3.2. Comparison of Independent Variables According to Retearing

Table 2 shows the results of the univariate analysis according to retear after ARCR. The significant factors were age, type of medical insurance, diabetes, initial tear size, and symptom duration (*p* < 0.05). The average age was significantly higher among patients with retears (64.7 ± 7.7 years) than among those without retears (*p* < 0.001). The proportion of retears was higher in patients enrolled in Medicaid than in those enrolled in NHI. The retear rates in patients with and without diabetes were 35% and 18%, respectively, showing a statistically significant difference (*p* < 0.001). The mean tear size was 29.5 mm for patients with retear and 19.8 mm for those without, showing a significant difference (*p* < 0.001). In addition, patients with retears had a longer duration of symptoms than those without, with an average duration of 2.4 years before surgery (*p* < 0.001).

### 3.3. Multivariate Logistic Regression Analysis

Table 3 presents the results of the multivariate logistic regression analysis of the factors associated with retear after ARCR in all patients. Age, occupation, type of medical insurance, diabetes, initial tear size, and symptom duration were significantly associated with retear risk. For each 1-year age increase, the retear risk significantly increased 1.04 times (*p* = 0.017, 95% CI, 1.01–1.07). Patients with manual labor had a significantly higher risk of retear than non-manual labor (*p* = 0.005, OR 1.95, 95% CI, 1.23–3.11). Medicaid insurance showed the strongest association with retear risk (*p* < 0.001, OR 4.34, 95% CI, 2.09–9.02). Moreover, for every 1 mm increase in tear size, the risk of retear was 1.06 times greater (*p* < 0.001, 95% CI, 1.05–1.08), and the longer the symptom duration, the higher the risk of retear (*p* < 0.001, OR 1.21, 95% CI, 1.09–1.33).

### 3.4. Influence of Tested Factors on the Retear Risk after ARCR According to Sex

Table 4 shows the influence of the tested factors on retear risk after ARCR according to the patients’ sex. There were 401 female patients (55.5%). The average age of male and female patients was 61.5 and 63.1 years, which was significantly different. However, there was no difference in the initial tear size and symptom duration between the two groups. In male patients, the retear rate was significantly different according to the type of medical insurance, diabetes, and initial tear size. In particular, the risk was 8.34 times higher in patients with Medicaid insurance than in those with NHI insurance (*p* < 0.001, OR 8.34, 95% CI, 2.78–25.07). In female patients, manual labor, initial tear size, and symptom duration were significantly associated with retear risk. Female patients who performed manual labor had a 2.23 times higher risk of retear than patients who performed non-manual labor (*p* = 0.025, OR 2.23, 95% CI, 1.11–4.49).

### 3.5. Influence of the Tested Factors on the Retear Risk after ARCR According to the Area of Residence

Table 5 shows the influence of factors on retear after ARCR according to the area of residence. In rural areas, factors that showed a significant association with retear after ARCR were occupation, type of medical insurance, diabetes, initial tear size, and symptom duration. Moreover, in urban areas, age, occupation, type of medical insurance, initial tear size, and symptom duration affected the risk of retear (*p* < 0.05). In both groups, the highest risk was observed in the Medicaid group. Especially in urban areas, elderly patients and those with manual labor had a higher risk of retear (*p* < 0.05).

## 4. Discussion

In this study, patients who underwent ARCR for a full-thickness rotator cuff tear had a mean age of 62.4 ± 8.0 years, mean tear size of 21.8 ± 12.5 mm, and mean symptom duration of 1.8 ± 1.7 years. In the comparison between patients with and without retears, there were statistically significant differences in age, type of medical insurance, diabetes, tear size, and symptom duration (*p* < 0.05). According to the multivariate logistic regression analysis, manual labor, type of medical insurance, diabetes, tear size, and symptom duration were associated with retear risk after ARCR after controlling for the influence of other factors.

Good anatomical and functional results after rotator cuff repair have previously been reported [5,6]. Patients with anatomical failure, such as retear or nonhealing, showed better results than before surgery; however, they had worse outcomes than patients without anatomical failure [1,6,8,30]. Moreover, revision ARCR is more difficult, shows poorer results, and has a higher rate of retear than primary ARCR. Therefore, it is important for orthopedic surgeons to make efforts to prevent rotator cuff retear; for this reason, many studies have been conducted to analyze risk factors for retear [1,6,8,10,30]. A systematic review by Zhao et al. [1] analyzed many papers and reported the risk factors for retear. According to this study, as in our study, among patient factors, age and diabetes were mentioned as representative risk factors. This is explained by poor tendon quality and low tendon healing efficacy in elderly patients [1,10]. In a study investigating the prevalence of posterosuperior rotator cuff tears, Park et al. [31] reported that physical exertion and diabetes were highly correlated. At the molecular level, hyperglycemia induces oxidative stress and cytokine production, leading to tendon degeneration and retear after ARCR.

The initial tear size influences the retear rate [1]. Patients with asymptomatic rotator cuff tears exist, but these tears increase in size and develop symptoms over time. Hence, periodic observation and radiological examination are important [32]. Our previous study reported an association between tear size and symptom duration [16]. This study also demonstrated a statistically significant association between symptom duration and rotator cuff retearing. In addition, age, symptom duration, and tear size are closely related, especially in situations of economic inequality. A social system and consensus should be established so that these patients can be diagnosed early and receive appropriate treatment.

Studies reporting that socioeconomic disparities can lead to chronic diseases have been published in various fields. Several studies have shown that there is an income-based difference in the incidence of diabetes, stroke, heart disease, and cancer [33,34,35,36]. These studies are valuable because social responsibility is required in situations where socioeconomic inequality is deepening, which is difficult to resolve at the individual level. Rotator cuff tear is the most representative chronic orthopedic disease. Moreover, it has a high association with age and manual labor [34]. In a cohort study of 393 patients with asymptomatic full-thickness rotator cuff tears, multivariate analysis found that pain increased with increasing comorbidity, lower educational level, and non-white race [29]. However, few studies have included socioeconomic factors in the outcomes of patients who underwent ARCR [2,37]. According to the studies of Rogers et al. [37] and Curry et al. [38], patients with Medicaid insurance experienced longer waiting times for physical therapy appointments and orthopedic consultations than patients with private insurance. In a study of 30,000 individuals by Chapman et al. [27], patients with Medicaid insurance from ethnic minorities were less likely to undergo surgery (OR 0.42, 95% CI, 0.34–0.50) and more likely to wait longer (OR 2.36, 95% CI, 2.02–2.70). In this study, the retear rate after ARCR was higher in the Medicaid group even after adjusting for other factors. In patients with Medicaid insurance, various factors are considered to contribute to retearing, such as comorbidities (e.g., diabetes), poor access to care, delayed diagnosis, delay in surgery, and inappropriate rehabilitation after surgery.

Furthermore, several studies have already demonstrated that the prevalence of rotator cuff tears is high in heavy labor workers who perform repetitive or excessive activities [39,40]. Work-related factors have been overlooked by orthopedic surgeons. Therefore, studies on the influence of these factors on failure after rotator cuff surgery are extremely rare. In this study, retearing was associated with manual labor in both univariate and multivariate analyses. However, in this study, it was not possible to conduct a clear investigation into the reasons for this association. In the future, studies on tissue quality related to worker-related tears should be conducted.

According to Razmjou et al. [4], sex differences are not simply biological but act as a social determinant in rotator cuff tears. These authors also reported that sex disparities affect mechanisms of injury, perceived disability, medication consumption, referral patterns, and surgical waiting times. In addition, it has been reported that repetitive activities act as a risk factor for rotator cuff tears in female compared to male patients. In this study, although there was no difference in retear rates between men and women, the risk factors for retearing were analyzed separately. The type of medical insurance and diabetes were risk factors in men, and manual labor was significant in women.

In addition, previous studies have reported that tear size is affected by symptom duration in rural areas and by manual labor in urban areas. In this study, retear rates were higher in patients with longer symptom duration in rural areas and in manual laborers in urban areas. This result is due to the correlation between tear size and retear rate. Additionally, delays in diagnosis due to poor healthcare access in rural areas may be related to symptom duration.

Although this study analyzed 10 years of data from a single hospital, it had limitations as a retrospective study. First, there are several factors related to retearing; however, as this was a retrospective study, data collection was limited. Thus, not all factors could be analyzed. Second, fatty degeneration or infiltration is an important variable but was not included in this study. However, as tear size and fatty changes are closely related, incorporating them as variables can confound the data.

## 5. Conclusions

Age, diabetes, initial tear size, and symptom duration significantly affect the risk of retear after ARCR. Socioeconomic factors, such as occupation and type of medical insurance, also affect the risk of retear after surgery. It is necessary to be aware of the various risk factors for retear after ARCR and to take an appropriate approach before and after surgery.

## Figures and Tables

**Table 1:** General characteristics of the participants.

		N	%
Sex	Female	401	55.5
	Male	322	44.5
Age (mean ± SD, range; years)		62.4 ± 8.0, 30–87	
Age group (years)	30–49	30	4.1
	50–69	549	75.9
	≥70	144	19.9
Occupation	Non-manual	254	35.1
	Manual	469	64.9
Education	≤Middle school	348	48.1
	≥High school	375	51.9
Area of residence	Urban	485	67.1
	Rural	238	32.9
Insurance type	NHI	681	94.2
	Medicaid	42	5.8
Obesity	No	393	54.4
	Yes	330	45.6
Diabetes	No	603	83.4
	Yes	120	16.6
Symptom duration (mean ± SD, range; years)		1.8 ± 1.7, 1–10	
Tear size (mean ± SD, range; mm)		21.8 ± 12.5, 5–49	

**Table 2:** Comparison of variables according to the occurrence of retears.

		No Retear		Retear		*p*-Value
		N	%	N	%	
Sex	Female	317	55.4	84	55.6	0.963
	Male	255	44.6	67	44.4	
Age		61.8, 8.0		64.7, 7.7		<0.001
Age group (years)	30–49	27	4.7	3	2.0	<0.001
	50–69	448	78.3	101	66.9	
	≥70	97	17.0	47	31.1	
Occupation	Non-manual	206	36.0	48	31.8	0.333
	Manual	366	64.0	103	68.2	
Education	≤Middle school	286	50.0	62	41.1	0.05
	≥High school	286	50.0	89	58.9	
Area of residence	Urban	383	67.0	102	67.5	0.891
	Rural	189	33.0	48	31.8	
Insurance type	NHI	549	96.0	132	87.4	<0.001
	Medicaid	23	4.0	19	12.6	
Obesity	No	313	54.7	80	53.0	0.703
	Yes	259	45.3	71	47.0	
Diabetes	No	494	86.4	109	72.2	<0.001
	Yes	78	13.6	42	27.8	
Tear size (mm)		19.8 ± 11.3		29.5 ± 13.7		<0.001
Symptom duration (years)		1.6 ± 1.5		2.4 ± 1.7		<0.001

**Table 3:** Results of multivariate logistic regression analysis on the factors associated with the risk of shoulder rotator cuff retear.

		OR	LL	UL	*p*-Value
Sex	Male	Reference			
	Female	0.81	0.52	1.26	0.352
Age		1.04	1.01	1.07	0.017
Occupation	Non-manual	Reference			
	Manual	1.95	1.23	3.11	0.005
Education	≥High school	Reference			
	≤Middle school	0.92	0.58	1.45	0.718
Area of residence	Urban	Reference			
	Rural	1.06	0.69	1.63	0.789
Insurance type	NHI	Reference			
	Medicaid	4.34	2.09	9.02	<0.001
Obesity	No	Reference			
	Yes	0.97	0.65	1.46	0.902
Diabetes	No	Reference			
	Yes	2.43	1.49	3.95	<0.001
Tear size		1.06	1.05	1.08	<0.001
Symptom duration		1.21	1.09	1.33	<0.001

**Table 4:** Influence of tested factors on rotator cuff retear risk according to patient sex.

		Male				Female			
		OR	LL	UL	*p*-Value	OR	LL	UL	*p*-Value
Age		1.04	1.00	1.09	0.072	1.03	0.99	1.07	0.186
Occupation	Non-manual	Reference				Reference			
	Manual	1.88	0.95	3.74	0.071	2.23	1.11	4.49	0.025
Education	≥High school	Reference				Reference			
	≤Middle school	0.98	0.50	1.92	0.964	1.05	0.55	1.99	0.886
Area of residence	Urban	Reference				Reference			
	Rural	1.09	0.55	2.13	0.811	1.12	0.63	2.00	0.698
Insurance type	NHI	Reference				Reference			
	Medicaid	8.34	2.78	25.07	<0.001	2.39	0.84	6.85	0.104
Obesity	No	Reference				Reference			
	Yes	1.04	0.55	1.95	0.905	1.06	0.61	1.83	0.845
Diabetes	No	Reference				Reference			
	Yes	5.69	2.83	11.43	<0.001	0.93	0.43	2.01	0.847
Tear size		1.06	1.03	1.08	<0.001	1.07	1.04	1.09	<0.001
Symptom duration		1.14	0.98	1.32	0.100	1.30	1.13	1.51	<0.001

**Table 5:** Influence of tested factors on the retear risk according to the area of residence.

		Rural				Urban			
		OR	LL	UL	*p*-Value	OR	LL	UL	*p*-Value
Sex	Male	Reference				Reference			
	Female	0.85	0.38	1.89	0.684	0.74	0.43	1.28	0.282
Age		1.02	0.97	1.08	0.372	1.05	1.01	1.08	0.015
Occupation	Non-manual	Reference				Reference			
	Manual	2.44	1.07	5.59	0.034	1.76	1.00	3.10	0.052
Education	≥High school	Reference				Reference			
	≤Middle school	0.81	0.36	1.82	0.606	0.98	0.56	1.71	0.951
Insurance type	NHI	Reference				Reference			
	Medicaid	7.96	1.48	42.72	0.015	3.80	1.69	8.55	0.001
Obesity	No	Reference				Reference			
	Yes	0.71	0.34	1.48	0.355	1.12	0.69	1.83	0.649
Diabetes	No	Reference				Reference			
	Yes	4.94	2.11	11.56	<0.001	1.62	0.88	3.00	0.123
Tear size		1.07	1.04	1.10	<0.001	1.06	1.04	1.08	<0.001
Symptom duration		1.23	0.95	1.60	0.120	1.19	1.07	1.33	0.002

## Data Availability

The data will be available upon reasonable request.
